# The role of histidines in amyloid β fibril assembly

**DOI:** 10.1002/1873-3468.12616

**Published:** 2017-04-03

**Authors:** Kristoffer Brännström, Tohidul Islam, Linda Sandblad, Anders Olofsson

**Affiliations:** ^1^ Department of Medical Biochemistry and BiophysicsUmeå UniversitySweden; ^2^ Department of Molecular BiologyUmeå UniversitySweden

**Keywords:** abeta, amyloid, fibril, histidine, stability, surface plasmon resonance

## Abstract

Low pH has a strong stabilising effect on the fibrillar assembly of amyloid β, which is associated with Alzheimer's disease. The stabilising effect is already pronounced at pH 6.0, suggesting that protonation of histidines might mediate this effect. Through the systematic substitution of the three native histidines in Aβ for alanines, we have evaluated their role in fibril stability. Using surface plasmon resonance, we show that at neutral pH the fibrillar forms of all His‐Ala variants are destabilised by a factor of 4–12 compared to wild‐type Aβ. However, none of the His‐Ala Aβ variants impair the stabilising effect of the fibril at low pH.

## Abbreviations

**Aβ**, Amyloid β

**K_D_**, dissociation constant

**Pnf**, potentiation factor

**SPR**, surface plasmon resonance

The self‐assembly of amyloid β (Aβ) into amyloid plaques is strongly linked to the development of Alzheimer's disease, which is the most common form of dementia and afflicts around 35 million people worldwide 1. Aβ is a 39–42 residue long peptide where the two forms Aβ_1–40_ and Aβ_1–42_ dominate. Aβ is derived from the proteolytic cleavage of the single transmembrane Aβ precursor protein by β‐ and γ‐secretase activity 2, 3. Depositions of amyloid fibrils seen as plaques as well as intracellular neurofibrillary tangles and neuronal loss are clinical hallmarks of Alzheimer's disease 4.

A delicate balance controls the difference between a native monomeric Aβ peptide and its pathological aggregated state, and just a small shift in this equilibrium can have detrimental effects 5, 6, 7, 8, 9, 10. Elucidation of the mechanisms that mediate the pathological self‐assembly is consequently of interest for developing therapeutic interventions against amyloid diseases.

The formation of Aβ fibrils requires an initial nucleation event, and fibril elongation involves the addition of monomers to an existing fibril. In analogy to all polymerisation processes, Aβ fibril formation is concentration dependent, and at a concentration of free monomers below the dissociation constant (K_D_), also known as the critical concentration, fibril formation cannot occur 11, 12. We have previously determined that the critical concentration for Aβ at neutral pH is around 100 nm for Aβ_1–40_ and slightly lower for Aβ_1–42_ 13. A similar value was also previously found by Hasegawa and co‐workers 14. However, the extracellular concentration of Aβ *in vivo* is around 1 nm for Aβ_1–40_ and even lower for the Aβ_1–42_ variant 15. The low concentration *in vivo* in combination with the significantly higher K_D_ has puzzled the field because fibrillar formation under these conditions should, in theory, be prohibited.

It has recently been shown that Aβ self‐assembly is highly dependent on pH and that a lowering of the pH significantly enhances the stability of the fibrils 11. The K_D_ for Aβ_1–40_ can be as much as 120 times lower at pH 4.5 than pH 7.4. This has several implications because it shows that Aβ assembly can indeed form at physiological Aβ concentrations and also strongly suggests that it occurs within intracellular compartments having a lower pH, such as endosomes or lysosomes 11. It is therefore of interest to elucidate the stabilising factors associated with fibril stability and the potentiating effect of low pH.

The strong pH dependency is likely caused by a change in charge through the protonation of certain amino acid side chains. The stabilising effect is pronounced already at pH 6.0, and within this range only the histidine side chains, having a pKa around 6.0 are significantly affected. It has also previously been shown that truncating the first 16 amino acids of the N‐terminus of the peptide results in a loss of the stabilising effect at low pH 11, which indicates that the N‐terminal part controls this mechanism. The Aβ sequence contains three histidines; all located in the N‐terminal part of the peptide at positions 6, 13 and 14. In the present work, we have evaluated how the stabilising effect of lowering the pH is affected in the Aβ_1–40_ variant by the systematic substitution of the three histidines for alanines. Using surface plasmon resonance (SPR), we studied the single mutation variants Aβ_1–40 H6A_, Aβ_1–40 H13A_ and Aβ_1–40 H14A_ as well as a variant where all three histidines have been exchanged (Aβ_1–40 H6A, H13A, H14A_).

Amyloid polymerisation follows a template‐dependent polymerisation process where monomers are incorporated onto the fibrillar ends. The stability of an amyloid fibril (in analogy to all binding interactions) is determined by the rate of association (k_(on)_) and the rate of dissociation (k_(off)_), which reflects how fast a monomer is incorporated onto the fibrillar end and how long it remains bound.

Using SPR, it is possible to monitor both the k_(on)_ and the k_(off)_ in a polymerisation reaction, such as the formation of amyloid. The method is based on the immobilisation of preformed fibrils onto a surface followed by the subsequent probing of free monomers. The Aβ monomers then incorporate onto the fibrillar ends and a continuous increase in the mass on the SPR surface can be monitored. Upon stopping the injection, while maintaining a flow of the running buffer, fibril dissociation into monomers can selectively be observed. Through monitoring both the association and the dissociation, the strength of the interaction, K_D_, reflecting the fibril stability, can be evaluated 13, 14, further discussed below.

Using SPR, we show that substituting any of the histidines results in significantly lower stability of the fibrils, at neutral pH, compared to the wild‐type peptide (Aβ_1–40 wt_). However, no single site mutant impaired the stabilising effect of low pH and, similar to Aβ_1–40 wt_, they all acquired more than a 200‐fold increase in fibril stability upon shifting the pH from 7.4 to 5.5. This suggests that histidine residues contribute to fibril stability at neutral pH but are not involved in the strong potentiating effect on fibril stability induced by low pH.

## Material and methods

### Preparation of monomeric Aβ peptides

Recombinant variants of Aβ_1–40_, Aβ_1–40 H6A_, Aβ_1–40 H13A_, Aβ_1–40 H14A_ and Aβ_1–40 H6A, H13A, H14A_ were obtained from AlexoTech (Umeå, Sweden). All lyophilised peptides were solubilised in 20 mm NaOH and separated by size‐exclusion chromatography (Superdex 10/300, GE Life Science, Uppsala, Sweden) in PBS with 2 mm EDTA, 5 mm NaOH and 150 mm NaCl. The pH of all purified peptide solutions was adjusted using a stock solution of phosphate or acetate buffer to give a final buffer concentration corresponding to 20 mm phosphate (pH 7.4) or acetate (pH 5.5) and 150 mm NaCl.

### Surface plasmon resonance (SPR)

The interaction study between the fibrillar form of Aβ and its monomeric form was performed using a BIA_core_® 3000 biosensor (GE Healthcare®, Uppsala, Sweden) equipped with a CM5 sensor chip (GE Healthcare®). First, the fibrillar form of Aβ was immobilised at a density of 3500 response units (RUs) on the dextran layer of the chip using standard amino coupling reagents at pH 4.0. All SPR experiments were performed at 25 °C at a flow rate of 20 μL·min^−1^ in either a 20 mm phosphate buffer (pH 7.4) or 20 mm acetate buffer (pH 5.5). All experiments were performed in the presence of 150 mm NaCl, which represents a physiological ion‐strength.

Due to the polymeric nature of amyloid fibrils, the covalent immobilisation on the sensor chip, likely only occurs in minor fraction of the accessible amino groups. This treatment is therefore not anticipated to influence the overall incorporation of monomers in a significant manner. However, the influence from using immobilised fibrils or the dextran layer cannot be fully excluded and should therefore be disclosed.

All sensograms were corrected for nonspecific interactions using a reference surface according to standard procedures 16.

### Transmission electron microscopy

A total volume of 4 μL of the different Aβ variants was absorbed for 2 min onto glow‐discharged carbon‐coated copper grids. Samples were washed in water and immediately stained in 50 μL of 1.5% uranyl acetate solution for 30 s. Negatively stained samples were examined on a JEM1230 transmission electron microscope (JEOL) at 80 kV. Transmission electron micrographs were recorded with a Gatan UltraScan 1000 2k × 2k pixel CCD camera using digitalmicropgraph™ software (Gatan, Pleasanton, CA, USA.).

## Results

### Kinetic analysis of fibril formation using SPR

In analogy to all molecular interactions, the stability of the binding between an Aβ monomer and the fibrillar end is dependent on both the rate of assembly k_(on)_ and the rate of dissociation k_(off)_.

**Figure 1:**
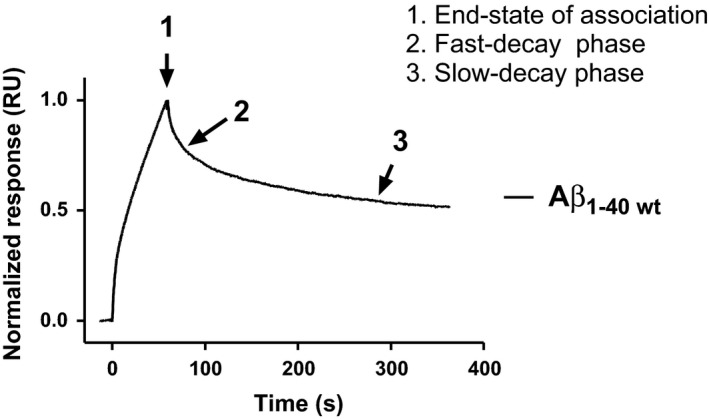
SPR analysis of Aβ fibril formation at pH 7.4. A representative sensogram of monomeric Aβ_1–40 wt_ when probed against immobilised Aβ_1–40 wt_ fibrils. The curve displays both the phase of association, where the end state of association is indicated (arrow 1), and the phase of dissociation‐phase, which frequently is divided into a fast phase (arrow 2) and a slow phase (arrow 3).

Probing free monomeric Aβ against immobilised fibrils using SPR is a frequently used technique for measuring the intrinsic kinetic properties of Aβ polymerisation 11, 14, 17, 18, 19. The SPR sensograms can be separated into an association phase (k_(on)_) and a dissociation phase ((k_(off)_). Figure 1 shows the result when immobilised Aβ_1–40 wt_ fibrils are probed with free Aβ_1–40 wt_ monomers.

The end state of the association phase is indicated by arrow 1 in Fig. 1.

Note that the dissociation phase exposes a biphasic curvature. This is highly representative and frequently observed when performing these kinds of experiments. The suggested mechanism behind this phenomenon is further discussed below. However, the initial fast phase of dissociation (indicated by arrow 2, Fig. 1) is much weaker than the second phase (indicated by arrow 3, Fig. 1) and does not contribute significantly to the fibril stability. All analyses of decay rates within this work are therefore exclusively focused on the slower second phase of dissociation.

Through a systematic exchange of the three histidine residues of Aβ, we will expose below their role within fibril formation with respect to association, dissociation and fibril stability.

### Kinetic analysis of the association rate, k_(on)_

**Figure 2:**
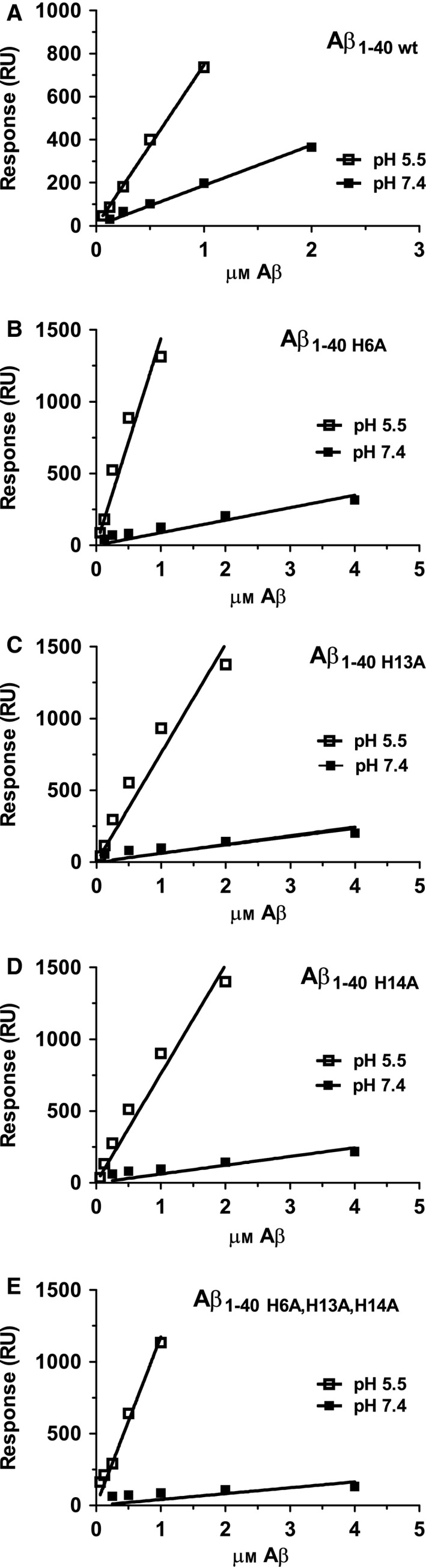
SPR analysis of the Aβ association rate as function of monomer concentration at pH 7.4 and 5.5. The monomeric Aβ variants at the indicated concentrations were probed towards their corresponding immobilised fibrils. The rates were acquired by measuring the end state of association after exactly 1 min of injection. pH 7.4 (filled squares), pH 5.5 (open squares). (A) Aβ_1–40 wt_. (B) Aβ_1–40 H6A_. (C) _Aβ1–40 H13A_. (D) Aβ_1–40 H14A_. (E) Aβ_1–40 H6A, H13A, H14A_.

Fibrils from recombinant variants of Aβ_1–40_, Aβ_1–40 H6A_, Aβ_1–40 H13A_, Aβ_1–40 H14A_ and Aβ_1–40 H6A, H13A, H14A_ were prepared by prolonged incubation in either PBS (pH 7.4) or acetate buffer pH 5.5 containing 150 mm NaCl. Fibrils immobilised on a CM5 chip were then probed with their monomeric counterpart at different concentrations of Aβ monomers and analysed by SPR. Samples were injected for exactly 60 s and the maximal value at the end state of each injection (indicated by arrow 1 in Fig. 1A) was plotted as a function of the monomeric concentration, Fig. 2. This generates an association rate at each point of measurement. A guideline is given in the figures to illustrate the linearity.

By comparing the rate between the different Aβ variants, it is possible to determine relative differences in k_(on)_.

At pH 7.4, all histidine mutants of Aβ had a k_(on)_ slightly slower than Aβ_1–40 wt_. The results are shown in Fig. 2 and the relative value for k_(on),_ (RA) for the different mutants versus Aβ_1–40 wt_ varies between 0.22 and 0.45 of Aβ_1–40 wt_ shown in Table 1.

**Table 1:** Relative change of association and dissociation rates of Aβ_1–40_ wt and the His‐Ala Aβ variants, and the potentiation of Aβ fibril formation as a function of lowering the pH. RA, Relative association; RD, Relative dissociation; PnF, Potentiation factor K_D(pH7.4)_/K_D(pH5.5)_

Aβ Variants	pH 7.4				pH 5.5				PnF
	RA	RD	RD/RA	K_D_ (nm)	RA	RD	RD/RA	K_D_ (nm)	
Aβ_1–40 wt_	1	1	1	100	1	1	1	1	100
Aβ_1–40 H6A_	0.45	1.92	4.27	427	1.94	2.78	1.43	1.43	299
Aβ_1–40 H13A_	0.31	2.22	7.16	716	1.01	2.13	2.11	2.11	339
Aβ_1–40 H14A_	0.34	2.70	7.94	794	1.01	2.22	2.19	2.19	363
Aβ_1–40 H6A, H13A, H14A_	0.22	2.70	12.27	1227	1.57	8.33	5.31	5.31	231

Interestingly, the corresponding k_(on)_ at pH 5.5 revealed that all Aβ His‐Ala variants, in analogy to Aβ_1–40 wt_, displayed a much faster rate of association as a function of a decrease in pH to 5.5. The degree of potentiation, indicated in Table 1, is similar to Aβ_1–40 wt_ and a significant difference in RA was only noted regarding Aβ_1–40 H6A_ and Aβ_1–40 H6A, H13A, H14A_, which displayed a 1.94 and 1.57 times higher RA when compared to Aβ_1–40 wt_.

### Kinetic analysis of dissociation rate

**Figure 3:**
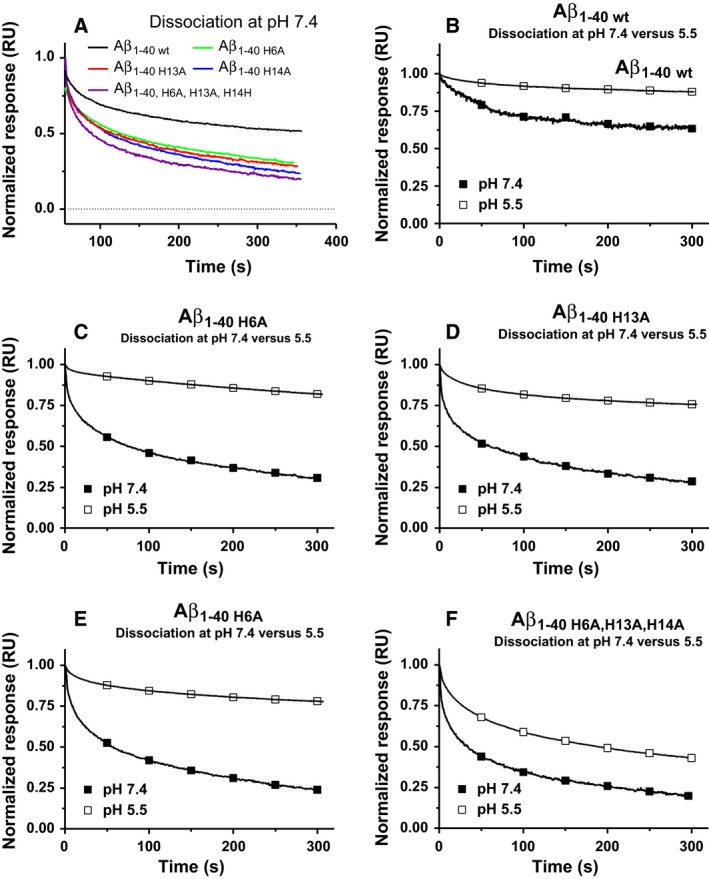
SPR analysis of Aβ fibrillar dissociation (k_(off)_). Sensograms showing the dissociation phase acquired subsequent to the injection of the monomeric Aβ variants at 2 μm onto their corresponding immobilised fibrils. (A) An overlay of all Aβ His‐Ala variants and Aβ_1–40 wt_ at pH 7.4. (B) Aβ_1–40 wt_ at pH 7.4 and 5.5. (C) Aβ_1–40 H6A_ at pH 7.4 and 5.5. (D) Aβ_1–40 H13A_ at pH 7.4 and 5.5. (E) Aβ_1–40 H14A_ at pH 7.4 and 5.5. (F) Aβ_1–40 H6A, H13A, H14A_ at pH 7.4 and 5.5.

In contrast to the association rate, k_on_, the rate of dissociation, k_off_, is independent of the concentration of monomers. Subsequent to probing, the immobilised fibrils with free monomers, the rate of dissociation can be seen as a decrease in the signal over time. Figure 3A shows a comparison between the rate of dissociation between the different fibrillar forms at pH 7.4. The result shows that all Aβ_1–40_ His‐Ala variants exhibit a higher rate of dissociation than Aβ_1–40 wt_. Figure 3B–F shows the intrinsic difference regarding each variant as a function of changing the pH from 7.4 to 5.5. The results show that Aβ_1–40 wt_, in accordance with previous results 11, is significantly stabilised by the decrease in pH. The results, however, also expose a significant stabilisation for all Aβ His‐Ala variants and a significantly slower phase of dissociation is observed upon lowering the pH to 5.5.

Because the latter part of the curve, representing the slower phase of dissociation (indicated by arrow 3 Fig. 1), strongly dominates the interaction strength, we have exclusively focused on this part, and no points before 100 s were included in the curve. The dissociation curve could then be fitted to a one exponential decay. The relative dissociation rate in relation to Aβ_1–40 wt_ (RD) for the different mutants is given in Table 1.

### Determination of the relative fibril stability

While a simple interaction between, for example, a receptor and ligand can easily be determined through saturation experiments, this cannot be done with polymers since they, per definition, cannot be saturated.

This problem can, however, be circumvented by defining the concentration of free monomers where the fibrils are at steady state, i.e. the concentration of free monomers where neither association nor dissociation can be detected. We have previously shown that the K_D_ for Aβ_1–40 wt_ at neutral pH is around 100 nm and around 1 nm at pH 5.5 11, 13. The K_D_ at neutral pH has also been investigated by Hasegawa and co‐workers 14 with similar results. All figures used here are therefore given as relative values to the K_D_ for Aβ_1–40 wt_ corresponding to 100 nm at neutral pH and 1 nm at pH 5.5.

Multiplying the relative RD/RA with the K_D_ for Aβ _1–40 wt_ will then give the K_D_ for the histidine derivatives.

For all of the evaluated His‐Ala Aβ variants, the substitutions destabilised the fibrillar conformation at neutral pH as indicated by a more rapid decay compared to Aβ _1–40 wt_ (Fig. 3A). The overall stability was decreased 4–12‐fold in all of the His‐Ala variants compared to Aβ_1–40 wt_. At low pH, all of the histidine to alanine substitutions displayed a pronounced stabilisation. The relative stabilisation was even stronger than observed in Aβ_1–40 wt_ and the potentiation ranged between 238 and 358 times simply by shifting the pH from 7.4 to 5.5. The dissociation relative to Aβ_1–40 wt_ and the overall potentiation factor (PnF) considering both the k_on_ and k_off_ is indicated in Table 1.

### Transmission electron microscopy verifies a fibrillar morphology

**Figure 4:**
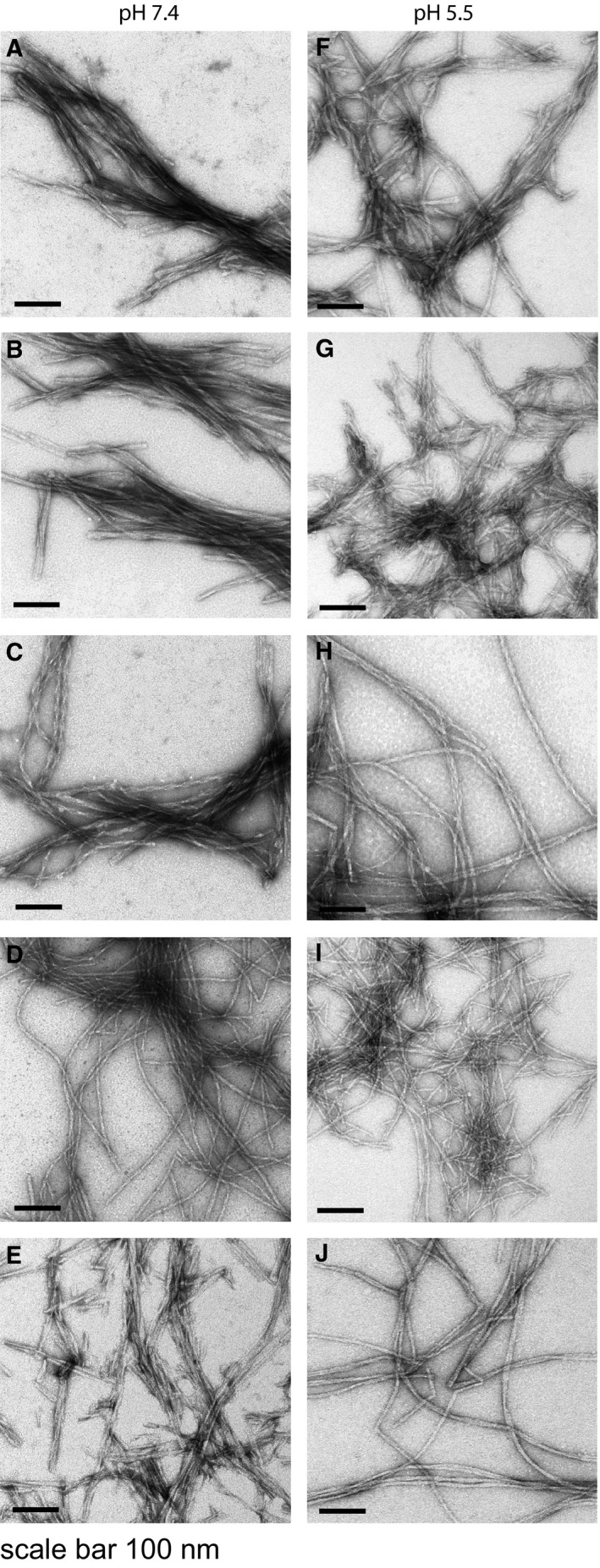
Negative stain transmission electron microscopy of Aβ assemblies. Evaluation of the morphology of the formed aggregates at pH 7.4 (A–E) and pH 5.5 (F–J). (A) Aβ_1–40 wt_. (B) Aβ_1–40 H6A_. (C) Aβ_1–40 H13A_. (D) Aβ_1–40 H14A_. (E) Aβ_1–40 H6A, H13A, H14A_. (F) Aβ_1–40 wt_. (G) β_1–40 H6A_. (H) Aβ_1–40 H13A_. (I) Aβ_1–40 H14A_. (J) Aβ_1–40 H6A, H13A, H14A_.

To verify the fibrillar morphology of the assembled peptides, we evaluated the acquired aggregates using negative‐stain transmission electron microscopy. Assemblies of Aβ_1–40 wt_ and the different Aβ His‐Ala variants, formed at pH 7.4 as well as 5.5, were analysed. The results showed that none of the variants were impaired in forming fibrils and all displayed a similar morphology as Aβ_1–40 wt_ fibrils, Fig. 4A–J.

## Discussion

A very delicate balance controls the self‐assembly of Aβ monomers *in vivo*, and also a very small shift in the equilibrium can have detrimental effects 6, 7, 8, 9, 20. Today, major efforts are being directed towards identifying methods that can prevent either Aβ excision from APP 3, 21, 22 or impair Aβ assembly 23, 24, 25, 26.

It is therefore of interest to understand the factors controlling fibril formation, as well as the properties of the acquired assemblies.

It has recently been shown how low pH has a strong potentiating effect on fibril stability 11. This finding also provides a possible explanation to how fibrils may form at the very low Aβ concentrations found in the human brain, which had previously been considered to be far below the critical concentration for Aβ fibril formation. This finding also strongly indicates that fibril formation occurs within the low pH compartments of the cell, such as endosomes and lysosomes.

From a mechanistic perspective, the effects of a change in pH on fibril formation are most likely due to protonation of amino acid side chains. Interestingly, a moderate lowering of the pH also has a strong effect where the potentiation factor already at pH 6.5 is around 20 times 11. This suggests that some functional groups, having a pKa, within this pH range, likely mediate the effect of pH. The histidine side chains in Aβ have a pKa corresponding to 6.0 27 and their protonation may therefore possibly represent a part of the mechanism.

In this work, we show how a single exchange of any of the three histidines in Aβ_1–40_ decreases the fibril stability at neutral pH relative to Aβ_1–40 wt_. Substitution of either His13 or His14 results in an approximately seven‐ to eightfold lower stability of the fibril, while the exchange of His6 results in an approximately fourfold decrease in fibril stability. Exchange of all three histidines results in an overall 12 times less stable fibrillar assembly. In all cases, we show that the destabilising effect is dependent on both a decreased rate of association and an increased rate of dissociation.

Somewhat surprisingly, all His‐Ala variants displayed a strong potentiation in binding strength upon lowering the pH from 7.4 to 5.5 and increased the fibrillar stability from 240–350 times. The result suggests that the histidine side chains do not mediate the potentiating effect.

It should also be noted that the relatively higher potentiation regarding the Aβ His‐Ala variants is mostly an effect of the decreased stability at neutral pH and does not imply a stronger fibril than Aβ_1–40 wt_ at low pH. At pH 5.5, all single‐substitution variants had 1.42–2.22‐fold lower stability compared to Aβ_1–40 wt_, while the fibrillar fold of the triple‐substituted variant had a 5.26‐fold lower stability.

Notably, the initial rapid phase of decay is also much more pronounced in the His‐Ala substitution variants. A biphasic behaviour of the dissociation curve has been reported previously and is suggested to be an effect of a dock and lock mechanism 28, 29, 30. This phenomenon implies that an initial poor association of the free monomer onto the fibrillar end first occurs, which is defined as the ‘dock’ phase. The peptide then adopts a more optimal orientation, with a stronger interaction between the newly incorporated monomer and the templating fibril, which is defined as the ‘lock’ phase.

A recent publication, however, suggests an alternative explanation where the initial low affinity interaction is due to a lateral assembly of monomers along already formed fibrils 31 and consequently does not reflect the interaction within the fibrillar end.

However, in terms of binding affinities, the initial rapid phase of dissociation only provides a small contribution to the overall binding. Upon analysis of the overall binding strength, the first phase has therefore been disregarded.

It is also of interest to discuss the role of histidines from a pathology perspective of AD. The role of fibrils as inert bystanders or active participants of the pathology is still a matter of debate and it is well known that prefibrillar assemblies, such as oligomers, exhibit a much higher cytotoxic effect than mature fibrils 32, 33, 34, 35. Factors that partly impair the assembly into fibrils may, instead, facilitate the formation of alternative assemblies. Aβ also has metal‐binding properties and the binding of both Zn^2+^ and Cu^2+^ has been shown 36, 37, 38, 39 which are both potent inducers of Aβ assembly 40, 41. However, the structure of the acquired aggregates differs significantly as compared to fibrils acquired in the absence of metals 36. Interestingly, a dysregulation of Zn^2+^, in the AD brain has also been assigned a pathophysiological role within AD where the increased levels of Zn^2+^ results in alternative Aβ assemblies 42. The binding of both Zn^2+^ and Cu^2+^ to Aβ involve all three histidines 43, 44 and, given present results showing that histidine residues affect the fibrillar stability, may suggest that metals may have the same effect.

Taken together, it can be concluded that the histidine residues of Aβ contribute to fibrillar stability at neutral pH but that their protonation is not a key event that can explain the strong potentiation in Aβ fibril stability that is induced as a function of low pH.

## Author contributions

KB performed the SPR experiments and LS performed the TEM analysis. AO, TI and KB directed the research and wrote the manuscript.
